# Multifunctional interface engineering enables efficient and stable inverted organic photovoltaics

**DOI:** 10.1038/s41467-025-60214-5

**Published:** 2025-05-26

**Authors:** Bowen Liu, Jian Qin, Qun Luo, Chang-Qi Ma

**Affiliations:** 1 i-Lab & Printable Electronics Research Center, Suzhou Institute of Nano-Tech and Nano-Bionics, Chinese Academy of Sciences, Suzhou, PR China; 2 College of Chemistry and Molecular Sciences, Henan University, Kaifeng, PR China

**Keywords:** Solar cells, Photovoltaics

## Abstract

The efficiency and light stability of inverted organic photovoltaics have been limited by the defects and photocatalytic reactivity of metal oxides transport layer. Here, authors discuss the recent progress and potential solutions for this technology to be put into production and industrialization.

## Teaser

Compared to conventional (p-i-n) organic photovoltaics (OPVs), inverted (n-i-p) OPVs hold promise for future commercial applications due to their advantages in printing process compatibility and environment robustness. The current bottleneck lies in the efficiency and light stability, which is closely related to the defects and the photocatalytic reactivity of metal oxides transport layer. This comment summarizes the recent progress on inverted OPV and outlines potential solutions to surmount the hurdles before the technology can be put into production.

## Structure of OPVs and performance features

**Fig. 1:**
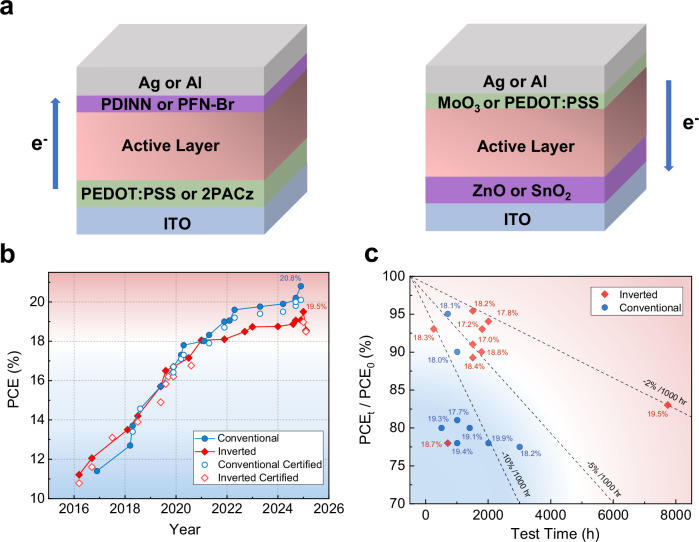
Comparison of conventional and inverted organic photovoltaics. **a** Device architecture of conventional and inverted organic photovoltaics (OPVs); **b** The efficiency development curve of inverted and conventional OPVs from 2016 to 2025; **c** the distribution of reported final power conversion efficiency (PCE) remaining (PCE_t_/PCE_0_) after aging for certain testing hours. Cells with an initial PCE over 17.5% and 17.0% for the conventional and inverted cells are chosen for comparison, and the initial PCE are listed in this figure. The dashed lines indicate different linear PCE degradation rates. The photovoltaic data and corresponding references are included in Supplementary Table 1 and 2.

Organic photovoltaics (OPVs) utilize a layered structure, where the key organic photoactive layer is sandwiched between a transparent metal oxide electrode (typically ITO) and a metal electrode (typically Ag or Al). Depending on the charge transport direction within the device, OPVs can be classified into conventional and inverted structures (Fig. 1a)^1^. In inverted devices, the bottom ITO electrode is typically coated with an electron transport layer (ETL) with low-work-function material, such as ZnO or SnO_2_ layer, acting as the cathode. At the same time, the top Ag or Al electrode is deposited on a high-work-function hole transport layer (HTL), such as MoO_3_, which serves as the anode. The conventional cells have an opposite layout, using typical PEDOT:PSS or 2-PACz as the HTL and PFN-Br or PDINN as the ETL, respectively. Notably, both the HTL and ETL play crucial roles in eliminating the interfacial barriers, improving charge injection and transport, and boosting the overall efficiency of the devices^2^.

Recent advancements in new non-fullerene acceptor molecules, morphology engineering of the photoactive layer, and the introduction of SAM molecules as the HTL have significantly boosted the development of conventional OPVs, with several laboratories achieving certified PCEs over 20%^3–5^. The efficiency breakthrough of conventional OPVs demonstrates that organic semiconductor-based photovoltaics can achieve efficiencies comparable to other types of cells, providing strong confidence in the commercialization potential of OPVs.

Unlike conventional counterparts, inverted OPVs use metal oxides such as ZnO, SnO_2_, and MoO_3_ as the charge transfer layer. Owing to the less thickness sensitivity of metal oxide interlayer, inverted OPVs are more compatible with different coating and printing methods, such as slot-die coating, blade coating, gravure printing, etc., making it the most promising candidate for large-area printing fabrication in the future^6^. Furthermore, inverted OPVs offer environmental adaptability and robustness owing to the use of high-work function HTL and metal electrode as the anode. Therefore, inverted OPVs are considered a promising direction for the industrialization of OPVs^7^. However, the efficiency of inverted OPVs still lags behind that of conventional OPVs, and the undesired photon decomposition of organic molecules on the surface of ZnO lowers the stability of inverted OPVs far from satisfactory

## Interfacial passivation in inverted organic solar cells

Since the first report of using oligothiophene-based A-D-A type non-fullerene acceptor (NFA) in OPV in 2015^8^, the PCE of OPVs has advanced rapidly in the last decade. Figure 1b presents the efficiency development trends of both conventional and inverted OPVs from 2016 to 2025. During the initial years (2016-2021), the efficiencies of these two different OPV types were quite similar, increasing from 11% to 18%, primarily due to the development of new NFA molecules. Since 2022, the PCE of conventional OPVs has increased much more rapidly than inverted cells. Although the strategies developed for conventional OPVs have been successfully used in inverted architectures, the efficiency gap remains. As of September 2024, the highest self-measured PCE of inverted OPVs reached 19.07%^9^, while the certified highest PCE was only 16.67%^10^ reported in 2020 (Fig. 1b). In addition to efficiency, stability is another crucial factor for OPVs. Figure 1c depicts the remaining PCEs of selected high-performance OPVs, with initial PCE over 17.5% for conventional cells and 17% for inverted cells, after aging for certain hours. Although the statistic data revealed that the inverted cells showed better stability than the conventional cells, the decay rate of inverted cells remains high for real application. The unsatisfactory stability of inverted cells is mainly due to the photocatalytic reactivities of ZnO, which leads to UV- and white light-induced decomposition of non-fullerene molecules^11,12^. Based on this understanding, various ZnO passivation strategies have been developed for the inverted cells. As latest examples, Liu et al.^13^ and Huanget et al.^14^ reported the use of in-situ derived inorganic SiO_x_N_y_ thin film to passivate the ZnO surface defects and the organic 3-(3,5-di-*tert*-butyl-4-hydroxyphenyl)propionic acid (BHT) to cap ZnO nanoparticles, respectively, both of which simultaneously improve device efficiency and stability. The OPVs containing inorganic SiO_x_N_y_ passivation layer exhibited a PCE of 18.55% (certified PCE of 18.49%) with an estimated T_80_ lifetime of 24,700 h under white light illumination^13^. While, the BHT@ZnO based cell showed an efficiency of 19.50% (certified efficiency of 18.97%) and a PCE maintaining of 81% after more than 7000 h of continuous operation (corresponding to an average decay rate of 2%/1000 h), marking the most efficient and stable inverted OPV reported till today (Fig. 1b,c).

In details, Huang et al. confirmed that photoexcitation of ZnO generates hydroxyl radicals and superoxide anions. The hydroxyl radicals selectively react with the NFA, causing NFA decomposition, while the superoxide radicals preferentially interact with the polymer donor, leading to the degradation of cells as well. With these, the authors developed the strategy of using BTH-capped ZnO nanoparticles, where BTH serves as a radical scavenger to suppress the undesired photon decomposition of organic molecules. They examined the interaction between BHT and ZnO and discovered that BHT can form COOZn complexes with ZnO through its carboxyl group, effectively passivating the surface oxygen vacancies of ZnO. Furthermore, the BHT molecules can burst the generated hydroxyl radicals and superoxide radicals. This enables achieving a record efficiency of 19.50% for inverted OPV based on a ternary system (PM6:BTP-eC9:o-BTP-eC9), approaching 20% in a conventional structure. This marks the first reported instance of an inverted OPV efficiency exceeding 19%. Besides, the stability of the BHT@ZnO-based devices is significantly improved under multiple conditions, including shelf stability and maximum power point (mpp) tracking of the cells in air and N_2_, as well as UV stability. More importantly, the authors reported a long actual mpp tracking for 7724 h, the longest test time among the stability tests reported so far, which showed an over 81% PCE remaining after aging.

This work represents a breakthrough in the efficiency and stability of organic photovoltaics (OPVs), achieving an impressive efficiency of over 19% in inverted OPVs and real-tested T_80_ close to 8000 h. This advancement substantially boosts confidence in the potential of inverted OPV structures, which have long been considered a promising alternative to renewable energy technology. Additionally, the authors propose a degradation mechanism in which hydroxyl radicals selectively interact with acceptor materials, while superoxide radicals preferentially react with polymer donors during degradation. This finding not only sheds light on the underlying processes of OPV decay but also provides critical guidance for enhancing the stability of these devices. In addition, in comparison with other surface treatment methods, the current strategy provides highly printable ZnO inks, which would simplify the device preparation process and make it highly suitable for roll-to-roll (R2R) printing in the future. Overall, this work offers invaluable directions for continuously developing high-performance and stable inverted OPVs and reinforces confidence in their future viability.

## Future studies for inverted OPV

In addition to further improving the efficiency of inverted OPV, we anticipate future research to focus more on enhancing stability and large-area production, with stability being the top priority. Factors causing the PCE decay of OPVs include light, heat, electric field, and water/oxygen. The latter can, in principle, be addressed through excellent encapsulation, while the negative influence of light, heat, and electric fields can only be solved by the innovative design of materials and device structures. Therefore, the PCE degradation caused by light, heat, and electric field is typically considered as intrinsic, determining the maximum lifetime of the cells, while the quality of encapsulation determines the actual achievement of the longest lifetime. Understanding the intrinsic degradation mechanisms of inverted OPVs would be crucial in improving their intrinsic stability of OPVs.

Although significant progress has been made on suppressing photo degradation of the cells, the UV-light-induced degradation behavior of inverted OPVs is still a critical issue that needs to be addressed. This is because of the sensitivity of ZnO to UV light. A solution to further stabilize the ZnO interface, especially under UV light illumination, or an alteration to ZnO that is UV-light insensitive, will be a key focus of future inverted OPV development. Preliminary studies indicated that SnO_2_ could be a good ETL for OPV^11^. However, the conductivity of the SnO_2_ layer and the remaining “burn-in” degradation indicate that further investigation is still needed. Overall, understanding the interfacial degradation of inverted OPVs under light, thermal, and electric fields and finding methods to suppress these degradation processes will be the key focus for stability studies of OPVs. In addition to solutions for improving the intrinsic stability of inverted OPVs, the development of low-cost encapsulation technologies to provide efficient barriers against water and oxygen is also essential for enhancing the overall lifetime of OPV. The encapsulation technology for OPV, which has been severely underexplored, serves as a crucial technological foundation for their commercial applications.

As the efficiency and stability of inverted OPVs continue to improve, the next critical challenge will be the realization of low-cost mass production. Recently, R2R-printed flexible inverted OPVs have been successfully achieved^15^. It is worth noting that device performance decreases significantly when the cell area increases from a few square millimeters to a few square centimeters. Also, high-efficiency inverted OPVs rely on ultra-thin interfacial layers for surface passivation and protection, which also presents a challenge for realizing R2R printing in the future. Unlike the interfacial layers, the direct modification of the ZnO, such as that developed by Huang et al.^14^, not only improves the ZnO preservation efficiency but also simplifies the device preparation process, which is conducive to commercial preparation in the future.

Overall, inverted OPVs are more promising for future commercial applications based on their advantages in large-area printing and better stability. At the same time, since the metal oxides used are usually inherently defective, further development of methods to passivate the metal oxides will be important to improve the efficiency and stability of inverted organic photovoltaics.

## Supplementary information

Supplementary Information

## Data Availability

The data that support the findings of this study are presented in the article or supplementary information.
