# Supplementary material for: Multifunctional interface engineering enables efficient and stable inverted organic photovoltaics
Source: Nat Commun. 2025 May 26;16:4880. doi: 10.1038/s41467-025-60214-5 (PMC12106598; doi:10.1038/s41467-025-60214-5)
Supplement: Supplementary file 1 — Supplementary Information [file 41467_2025_60214_MOESM1_ESM.pdf]

Supplementary Information:

Multifunctional interface engineering enables efficient and stable inverted organic photovoltaics

Bowen Liu[1,2], Jian Qin[1], Qun Luo[1*], Chang-Qi Ma[1*]

[1] *i*-Lab & Printable Electronics Research Center, Suzhou Institute of Nano-Tech and Nano-Bionics, Chinese Academy of Sciences, Ruoshui Road 398, SEID, SIP, Suzhou 215123, P. R. China

[2] College of Chemistry and Molecular Sciences, Henan University, Kaifeng 475004, P. R. China

* Corresponding author: Qun Luo, email: qluo2011@sinano.ac.cn; Chang-Qi Ma, email: cqma2011@sinano.ac.cn

Supplementary Table 1. Summary PCE of reported organic solar cells

Year	Active layer	PCE(%)	Certified PCE (%)	Structure	Ref.
2016	PBDB-T:ITIC	11.21	10.78	Inverted	1
2016	PBDB-T:IT-M	12.05	11.60	Inverted	2
2017	PBDBT-SF:IT-4F	13.10	13.10	Inverted	3
2018	PM6:IT-4F	13.50	-	Inverted	4
2018	P2:IT-4F	14.20	13.90	Inverted	5
2019	PM6:Y6	15.70	14.90	Inverted	6
2019	PBDB-TF:BTP-4Cl	16.50	15.83	Inverted	7
2019	PM6:Y6:$PC_{61}BM$	16.50	16.20	Inverted	8
2019	PM6:Y6:3TP3T-4F	16.70	16.20	Inverted	9
2020	PM6 : Y18 : $PC_{71}BM$	17.11	16.76	Inverted	10
2020	PM6:Y6:$PC_{71}BM$	17.15	-	Inverted	11
2021	PM6:L8-BO	18.05	-	Inverted	12
2021	PM6:PB2F:BTP-eC9	18.10	-	Inverted	
2021	PM6:PB2F:BTP-eC9	18.10	-	Inverted	13
2022	PM6:L8-BO-F:Y6-BO	18.49	-	Inverted	14
2023	PM6:PM7-Si:BTP-eC9	18.73	-	Inverted	15
2024	PM6:L8-BO:BTP-ec9	18.75	-	Inverted	16

**2024**	**PM6:D18:L8-BO**	**18.87**	**-**	**Inverted**	**17**
**2024**	**PM6:L8-BO:BTP-S10**	**19.06**	**-**	**Inverted**	**18**
**2024**	**PM6:BTP-eC9**	**19.07**	**-**	**Inverted**	**19**
**2024**	**PM6:BTP-eC9:o-BTP-eC9**	**19.47**	**18.97**	**Inverted**	**20**
**2025**	**PM6:L8-BO**	**18.55**	**18.49**	**Inverted**	**21**
**2016**	**J71: ITIC**	**11.40**	**-**	**Conventional**	**22**
**2018**	**PTQ10: IDIC**	**12.70**	**-**	**Conventional**	**23**
**2018**	**PBDB-TF:IT-4F**	**13.70**	**13.40**	**Conventional**	**24**
**2018**	**PBDB-TF:IT-4F**	**14.57**	**14.57**	**Conventional**	**25**
**2019**	**PM6:Y6**	**15.70**	**-**	**Conventional**	**6**
**2019**	**PBDB-TF:BTP-4Cl-12**	**17.00**	**16.70**	**Conventional**	**26**
**2020**	**PM6:Y6**	**17.30**	**17.10**	**Conventional**	**27**
**2020**	**PBDB-TF:BTP-eC9**	**17.80**	**17.30**	**Conventional**	**28**
**2021**	**PM6:PM7:Y6:$PC_{71}BM$**	**18.07**	**-**	**Conventional**	**29**
**2021**	**PM6:L8-BO**	**18.32**	**17.90**	**Conventional**	**12**
**2021**	**PBQx-TF:eC9-2Cl:F-BTA3**	**19.00**	**18.70**	**Conventional**	**30**
**2022**	**PTQ10:BTP-FTh:IDIC**	**19.05**	**-**	**Conventional**	**31**
**2022**	**PM6:D18:L8-BO**	**19.60**	**19.20**	**Conventional**	**32**
**2023**	**PM6:BTP-eC9:BTP-S16:BTP-S17**	**19.76**	**19.41**	**Conventional**	**33**
**2024**	**PM6:BTP-eC9:o-BTP-eC9**	**19.90**	**19.50**	**Conventional**	**34**
**2024**	**PM6:L8-BO: L8-ThCl**	**20.10**	**20.00**	**Conventional**	**35**
**2024**	**D18:Z8:L8-BO**	**20.20**	**19.80**	**Conventional**	**36**
**2024**	**D18-Cl:BTP-4F-P2EH**	**20.80**	**20.10**	**Conventional**	**37**

Supplementary Table 2. Summary PCE_t/PCE_0 of reported organic solar cells.

Year	Structure	Device structure	PCE_0 (%)	PCE_t/PCE_0 (%)	Test Time(h)	Ref.
2021	Inverted	ITO/ZnO/NDI-B/PBDB-TF:BTP-eC9/MoO_x/Al	17.00	93	1800	38
2021	Inverted	ITO/ZnO/PET/PM6:L8-BO/ MoO_x/Al	17.02	91	1500	39
2024	Inverted	ITO/ZnO/PhTMABr/PM6:L8-BO:BTP-ec9/MoO_3/Ag	18.75	90	1780	16
2024	Inverted	ITO/ZnO/PM6:PM7-Si:BTP-eC9/Br-2PACz/MoO_3/Ag	18.73	78	700	15
2024	Inverted	ITO/ZnO:BA/PBDB-TF:HDO-4Cl:BTP-eC9/MoO_3/Ag	18.40	89	1500	40
2022	Inverted	ITO/ZnO/NMA/D18:N3/MoO_3/Ag	18.20	95	1500	41
2021	Inverted	ITO/NDI-B/PM6:BTP-eC9/MoO_x/Al	17.23	93	1800	42
2024	Inverted	ITO/ZnO/NMA-C0/ PM6:L8-BO/MoO_x/Ag	18.31	93	250	43
2024	Inverted	ITO/ZnO/PM6:BTP-eC9:o-BTP-eC9/MoO_x/Ag	19.47	82	7724	34
2025	Inverted	ITO/ZnO/PM6:L8-BO/MoO_x/Ag	17.77	94	2000	21
2021	Conventional	ITO/PEDOT:PSS/PM6:PM7:Y6:$PC_{71}BM$/PFNDI-Br/Ag	17.70	81	1000	29
2023	Conventional	ITO/PEDOT:PSS/PM6:DY-BO/PDINN/Ag	18.00	90	1000	44
2024	Conventional	ITO/PEDOT:PSS/ PM6:BTP-eC9/CIM/Ag	19.27	80	500	45
2023	Conventional	ITO/PEDOT:PSS/ PM6:PY-1S1Se:PY-2Cl PFN-Br/Ag.	18.20	78	3000	46
2024	Conventional	ITO/Poly-2PACz /PM6:PTQ10:L8-BOBCP/Ag	19.10	80	1400	47
2024	Conventional	ITO/PEDOT:PSS/PM6:BTP-eC9:Y6-1O:PC71BM/PNDIT-F3N/Ag	19.35	78	1000	48
2024	Conventional	ITO/2PACZ/PM6:L8-BOX:Tri-V/PNDIT- F3N/Ag	19.86	78	2000	49
2024	Conventional	ITO-Cl/PM6:SMAs/PNDIT-F3N/Ag	18.09	95	700	50

Supplementary References

1. Zhao, W., *et al.* Fullerene-Free Polymer Solar Cells with over 11% Efficiency and Excellent Thermal Stability. *Adv. Mater.* **28**, 4734-4739 (2016).
2. Li, S., *et al.* Energy-Level Modulation of Small-Molecule Electron Acceptors to Achieve over 12% Efficiency in Polymer Solar Cells. *Adv. Mater.* **28**, 9423-9429 (2016).
3. Zhao, W., *et al.* Molecular Optimization Enables over 13% Efficiency in Organic Solar Cells. *J. Am. Chem. Soc.* **139**, 7148-7151 (2017).
4. Fan, Q., *et al.* Synergistic effect of fluorination on both donor and acceptor materials for high performance non-fullerene polymer solar cells with 13.5% efficiency. *Sci. China Chem.* **61**, 531-537 (2018).
5. Li, S., *et al.* A Wide Band Gap Polymer with a Deep Highest Occupied Molecular Orbital Level Enables 14.2% Efficiency in Polymer Solar Cells. *J. Am. Chem. Soc.* **140**, 7159-7167 (2018).
6. Yuan, J., *et al.* Single-iunction organic solar cell with over 15% efficiency using fused-ring acceptor with electron-deficient core. *Joule* **3**, 1140-1151 (2019).
7. Cui, Y., *et al.* Over 16% efficiency organic photovoltaic cells enabled by a chlorinated acceptor with increased open-circuit voltages. *Nat. Commun.* **10**, 2515 (2019).
8. Yu, R., *et al.* Improved Charge Transport and Reduced Nonradiative Energy Loss Enable Over 16% Efficiency in Ternary Polymer Solar Cells. *Adv. Mater.* **31**, 1902302 (2019).
9. Song, J., *et al.* Ternary Organic Solar Cells with Efficiency >16.5% Based on Two Compatible Nonfullerene Acceptors. *Adv. Mater.* **31**, 1905645 (2019).
10. Zhu, C., *et al.* Tuning the electron-deficient core of a non-fullerene acceptor to achieve over 17% efficiency in a single-junction organic solar cell. *Energy Environ. Sci.* **13**, 2459-2466 (2020).
11. Cui, M., *et al.* A Cost-Effective, Aqueous-Solution-Processed Cathode Interlayer Based on Organosilica Nanodots for Highly Efficient and Stable Organic Solar Cells. *Adv. Mater.* **32**, 2002973 (2020).
12. Li, C., *et al.* Non-fullerene acceptors with branched side chains and improved molecular packing to exceed 18% efficiency in organic solar cells. *Nature Energy* **6**, 605-613 (2021).
13. Yu, R., *et al.* Efficient interface modification via multi-site coordination for improved efficiency and stability in organic solar cells. *Energy Environ. Sci.* **15**, 822-829 (2022).
14. Huang, Q., *et al.* Simultaneous improvement of efficiency and stability of inverted organic solar cell via composite hole transport layer. *J. Mater. Chem. A* **10**, 23973-23981 (2022).
15. Lin, Y., *et al.* 18.73% efficient and stable inverted organic photovoltaics featuring a hybrid hole-extraction layer. *Mater. Horizons* **10**, 1292-1300 (2023).
16. Xin, Y., *et al.* Multiarmed Aromatic Ammonium Salts Boost the Efficiency and Stability of Inverted Organic Solar Cells. *J. Am. Chem. Soc.* **146**, 3363-3372 (2024).

17. Wu, J., *et al.* Beyond Conventional Enhancements: Self-Organization of a Buffer Material on Tin Oxide as a Game-Changer for Improving the Performance of Inverted Organic Solar Cells. *Small* **20**, 2404066 (2024).
18. Nugraha, M.I., *et al.* Over 19% Efficient Inverted Organic Photovoltaics Featuring a Molecularly Doped Metal Oxide Electron-Transporting Layer. *Adv. Mater.* **36**, 2310933 (2024).
19. Suo, Z., *et al.* A Water Solution Processed Hybrid Electron Transport Layer Simultaneously Enhances Efficiency and Stability in Inverted Structure Organic Solar Cells. *Adv. Funct. Mater.* **n/a**, 2409699 (2024).
20. Huang, J., *et al.* 19.5% Inverted organic photovoltaic with record long-lifetime via multifunctional interface engineering featuring radical scavenger. *Nat. Commun.* **15**, 10565 (2024).
21. Liu, B., *et al.* Inverted organic solar cells with an in situ-derived SiOxNy passivation layer and power conversion efficiency exceeding 18%. *Nat. Photon.* **19**, 195-203 (2025).
22. Bin, H., *et al.* 11.4% Efficiency non-fullerene polymer solar cells with trialkylsilyl substituted 2D-conjugated polymer as donor. *Nat. Commun.* **7**, 13651 (2016).
23. Sun, C., *et al.* A low cost and high performance polymer donor material for polymer solar cells. *Nat. Commun.* **9**, 743 (2018).
24. Li, W., *et al.* A High-Efficiency Organic Solar Cell Enabled by the Strong Intramolecular Electron Push–Pull Effect of the Nonfullerene Acceptor. *Adv. Mater.* **30**, 1707170 (2018).
25. Zheng, Z., *et al.* A Highly Efficient Non-Fullerene Organic Solar Cell with a Fill Factor over 0.80 Enabled by a Fine-Tuned Hole-Transporting Layer. *Adv. Mater.* **30**, 1801801 (2018).
26. Cui, Y., *et al.* Organic photovoltaic cell with 17% efficiency and superior processability. *National science review* **7**, 1239-1246 (2020).
27. Liu, L., *et al.* Graphdiyne Derivative as Multifunctional Solid Additive in Binary Organic Solar Cells with 17.3% Efficiency and High Reproductivity. *Adv. Mater.* **32**, 1907604 (2020).
28. Cui, Y., *et al.* Single-Junction Organic Photovoltaic Cells with Approaching 18% Efficiency. *Adv. Mater.* **32**, 1908205 (2020).
29. Zhang, M., *et al.* Single-layered organic photovoltaics with double cascading charge transport pathways: 18% efficiencies. *Nat. Commun.* **12**, 309 (2021).
30. Cui, Y., *et al.* Single-Junction Organic Photovoltaic Cell with 19% Efficiency. *Adv. Mater.* **33**, 2102420 (2021).
31. Chong, K., *et al.* Realizing 19.05% Efficiency Polymer Solar Cells by Progressively Improving Charge Extraction and Suppressing Charge Recombination. *Adv. Mater.* **34**, 2109516 (2022).
32. Zhu, L., *et al.* Single-junction organic solar cells with over 19% efficiency enabled by a refined double-fibril network morphology. *Nat. Mater.* **21**, 656-663 (2022).

33. Chen, T., *et al.* Compromising Charge Generation and Recombination of Organic Photovoltaics with Mixed Diluent Strategy for Certified 19.4% Efficiency. *Adv. Mater.* **35**, 2300400 (2023).
34. Fu, J., *et al.* Rational molecular and device design enables organic solar cells approaching 20% efficiency. *Nat. Commun.* **15**, 1830 (2024).
35. Chen, C., *et al.* Molecular interaction induced dual fibrils towards organic solar cells with certified efficiency over 20%. *Nat. Commun.* **15**, 6865 (2024).
36. Jiang, Y., *et al.* Non-fullerene acceptor with asymmetric structure and phenyl-substituted alkyl side chain for 20.2% efficiency organic solar cells. *Nature Energy* **9**, 975-986 (2024).
37. Zhu, L., *et al.* Achieving 20.8% organic solar cells via additive-assisted layer-by-layer fabrication with bulk structure and improved optical management. *Joule* **8**, 3153-3168 (2024).
38. Liao, Q., *et al.* Highly Stable Organic Solar Cells Based on an Ultraviolet-Resistant Cathode Interfacial Layer. *CCS Chemistry* **4**, 938-948 (2022).
39. Liu, B., *et al.* Simultaneously Achieving Highly Efficient and Stable Polymer:Non-Fullerene Solar Cells Enabled By Molecular Structure Optimization and Surface Passivation. *Adv. Sci.* **9**, 2104588 (2022).
40. Wang, Y., *et al.* New Method for Preparing ZnO Layer for Efficient and Stable Organic Solar Cells. *Adv. Mater.*, 2208305 (2022).
41. Li, S., *et al.* Achieving over 18% Efficiency Organic Solar Cell Enabled by a ZnO-Based Hybrid Electron Transport Layer with an Operational Lifetime up to 5 Years. *Angew. Chem. Int. Ed.* **134**, e202207397 (2022).
42. Liao, Q., *et al.* Highly Stable Organic Solar Cells Based on an Ultraviolet-Resistant Cathode Interfacial Layer. *CCS Chemistry* **4**, 938-948 (2021).
43. Suo, Z., *et al.* An efficient interface modification material for improved efficiency and stability in inverted organic solar cells. *Mater. Chem. Front.* **8**, 562-566 (2024).
44. Sun, C., *et al.* Dimerized small-molecule acceptors enable efficient and stable organic solar cells. *Joule* **7**, 416-430 (2023).
45. Mai, T.L.H., *et al.* Open-air, green-solvent processed organic solar cells with efficiency approaching 18% and exceptional stability. *Energy Environ. Sci.* **17**, 7435-7444 (2024).
46. Sun, R., *et al.* 18.2%-efficient ternary all-polymer organic solar cells with improved stability enabled by a chlorinated guest polymer acceptor. *Joule* **7**, 221-237 (2023).
47. Ren, Z., *et al.* Efficient and stable organic solar cells enabled by a poly(carbazole phosphonic acid) hole transporter. *Sci. China Chem.* **67**, 1941-1945 (2024).
48. Zhang, W., *et al.* A high-efficiency and stable organic solar cell with balanced crystallization kinetics. *Energy Environ. Sci.* **17**, 2182-2192 (2024).
49. Song, J., *et al.* Non-halogenated Solvent-Processed Organic Solar Cells with Approaching 20 % Efficiency and Improved Photostability. *Angew. Chem. Int. Ed.* **63**, e202404297 (2024).
50. Zhao, J., Yang, X., Shao, Y., Sun, R. & Min, J. Simultaneously improving efficiency and stability of organic solar cells by enhancing molecular crystallinity and intermolecular interactions. *Science China Materials* (2024).
